# Transcriptome study digs out *BMP2* involved in adipogenesis in sheep tails

**DOI:** 10.1186/s12864-022-08657-8

**Published:** 2022-06-21

**Authors:** Meilin Jin, Xiaojuan Fei, Taotao Li, Zengkui Lu, Mingxing Chu, Ran Di, Xiaoyun He, Xiangyu Wang, Caihong Wei

**Affiliations:** 1 Institute of Animal Sciences, Chinese Academy of Agricultural Sciences, Beijing, China; 2 College of Animal Science and Technology, China Agricultural University, Beijing, China; 3 Lanzhou Institute of Husbandry and Pharmaceutical Sciences, Chinese Academy of Agricultural Sciences, Lanzhou, Gansu, China

**Keywords:** Fat-tailed sheep, Transcriptome, Preadipocytes, *BMP2*, Adipogenesis

## Abstract

**Background:**

Hu sheep and Tibetan sheep in China are characterized by fat tails and thin tails, respectively. Several transcriptomes have been conducted in different sheep breeds to identify the differentially expressed genes (DEGs) underlying this trait. However, these studies identified different DEGs in different sheep breeds.

**Results:**

Hence, RNA sequencing was performed on Hu sheep and Tibetan sheep. We obtained a total of 45.57 and 43.82 million sequencing reads, respectively. Two libraries mapped reads from 36.93 and 38.55 million reads after alignment to the reference sequences. 2108 DEGs were identified, including 1247 downregulated and 861 upregulated DEGs. GO and KEGG analyses of all DEGs demonstrated that pathways were enriched in the regulation of lipolysis in adipocytes and terms related to the chemokine signalling pathway, lysosomes, and glycosaminoglycan degradation. Eight genes were selected for validation by RT–qPCR. In addition, the transfection of *BMP2* overexpression into preadipocytes resulted in increased *PPAR-γ* expression and expression. *BMP2* potentially induces adipogenesis through *LOX* in preadipocytes. The number of lipid drops in *BMP2* overexpression detected by oil red O staining was also greater than that in the negative control.

**Conclusion:**

In summary, these results showed that significant genes (*BMP2*, *HOXA11*, *PPP1CC* and *LPIN1*) are involved in the regulation of adipogenesis metabolism and suggested novel insights into metabolic molecules in sheep fat tails.

**Supplementary Information:**

The online version contains supplementary material available at 10.1186/s12864-022-08657-8.

## Introduction

Sheep are one of the main livestock resources around the world, consuming the produce meat, milk, wool, and fur [1]. Fat-tailed sheep make up a quarter of the world's sheep population. In China, there are more than 98 indigenous sheep breeds, 80% of which are fat-tailed sheep. Fat tails are also food for humans. However, with the increase in people's life standards, mutton consumption has been increasing, and the utilization rate of fat in sheep is lower. The intensity of fat deposition in the sheep tail is higher than that in the rest of the body [2]. Conversely, excessive fat deposition affects the feed conversion rate of sheep, which increases the cost of farmers' breeding. In production, a large amount of fat tail is directly discarded, forming a considerable amount of waste. Reducing fat deposition can produce leaner meat. It is important for the sheep industry for pastures to increase economic profits [3]. Consequently, this provides a better understanding of the molecular mechanism of lipogenesis in sheep breeding and control of carcass fat [4].

In recent years, several genomic approach studies have been conducted to reveal the important genes of the fat tail phenotype in different sheep breeds [5]. *BMP2* and *PDGFD* are likely potential genes related to fat deposition in the tails of sheep [6, 7]. In addition to these data, potential genes involved in the development of the fat tail were analysed in fat-tailed breeds. Mohammad and colleagues [8] identified some genes as important candidates associated with fat-tailed deposition of sheep, such as *HOXA10*, *ACSS2*, *ELOVL6*, *BMP6* and *FABP4*. Comparing Lanzhou fat-tailed sheep with two others thin-tailed sheep by using RNA-seq, *CREB1*, *WDR92* and *ETAA1* are potentially associated with fat tail development [5]. These results have provided abundant information for elucidating the genetic mechanism of fat deposition among these breeds. Nonetheless, the complex genetic factors associated with fat tail development also need further study. Therefore, it is important to study fat deposition in Hu sheep and Tibetan sheep.

The transcriptome provides an opportunity to reveal the underlying mechanisms of fat deposition in sheep [8]. In addition, some studies have compared various fat deposition in the transcriptomes of different species, such as pigs [9], cattle [10] and humans [11]. Therefore, RNA-Seq was used to identify DEGs, and regulatory pathways related to sheep fat deposition between Hu sheep and Tibetan sheep breeds. Combined with our previous analysis results [12–14], *BMP2* overexpression in preadipocytes provides a theoretical basis for the genetic improvement of sheep tail fat.

## Results

### RNA-seq data summary

**Fig. 1:**
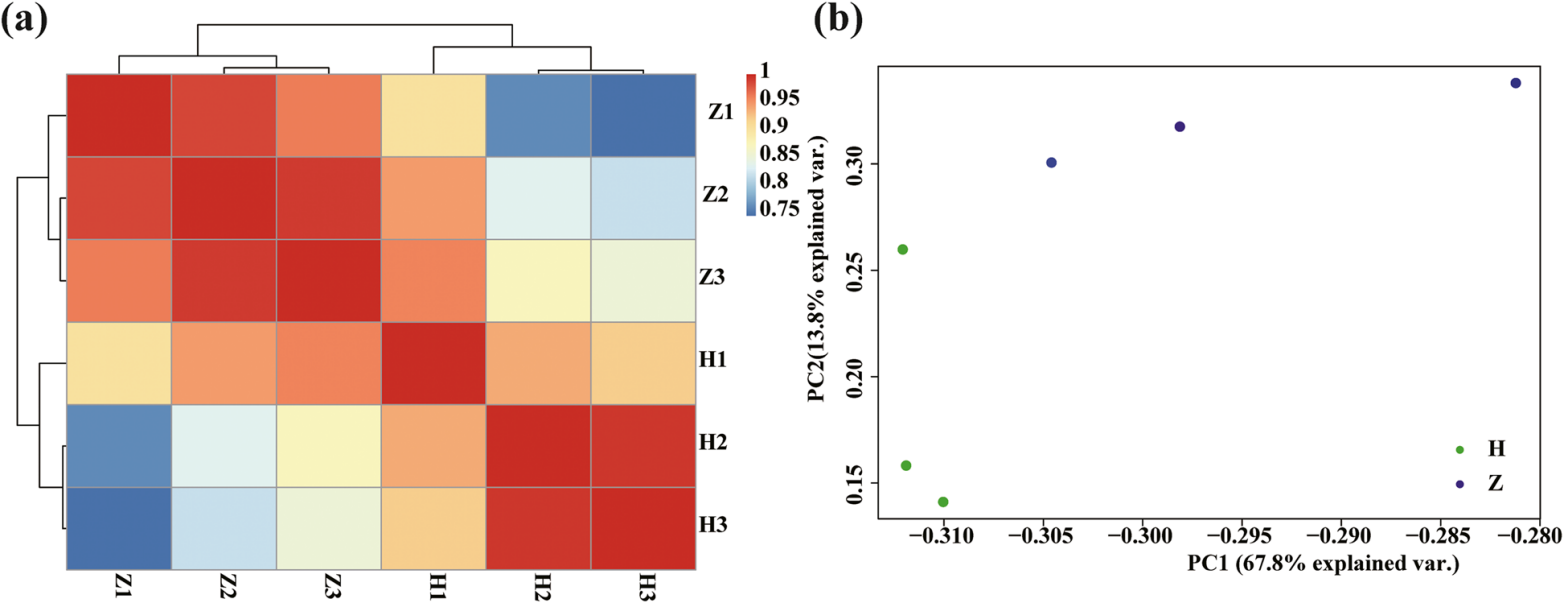
**a** Heatmap of gene expression of adipose tissue in the Hu sheep (H1, H2 and H3) and Tibetan sheep (Z1, Z2 and Z3) tails; **b** PCA of gene expression of adipose tissue in the Hu sheep (H) and Tibetan sheep tails (Z)

Two cDNA libraries of adipose tissue in tails from Hu sheep and Tibetan sheep were sequenced. A total of 45.57 million paired-end raw reads were obtained from six sheep samples. The size of each sample ranged from 43.02 to 44.65 million per sample. HISAT2 and Bowtie2 tools compared 86.03 and 65.41% clean reads with reference genomes, respectively. Moreover, clean reads were mapped to the reference genome (*Ovis_v3.1*). The RNA-sequencing and mapping information of the samples is shown in Table 1. These data were preliminarily analysed. The sample correlation was performed with the Pearson correlation coefficients based on gene expression levels, and these coefficients were reflected in the form of a heatmap (Fig. 1).

**Table 1:** Descriptive statistics of sequence quality and mapping rate from Hu sheep and Tibetan sheep

Breed	Raw reads (Million)	Trimmed reads	HISAT2 total mapped reads (%)	Bowtie2 total mapped reads (%)	HISAT2 uniquely mapped reads (%)	Bowtie2 uniquely mapped reads (%)
Z1	43.82	43.12	36.93(85.64)	27.32(63.36)	24.20(56.13)	22.07(51.18)
Z2	43.82	43.22	37.15(85.97)	27.63(63.93)	23.84(55.16)	22.25(51.48)
Z3	43.82	43.02	37.02(86.06)	27.70(64.38)	23.70(55.10)	22.01(51.16)
H1	43.82	43.12	37.27(86.43)	27.63(64.07)	23.13(53.63)	21.31(49.41)
H2	45.57	44.65	38.31(85.80)	30.16(67.55)	19.85(44.46)	21.37(47.85)
H3	45.57	44.68	38.55(86.29)	30.90(69.16)	19.80(44.31)	21.06(47.13)

Z1, Z2, and Z3 represent three biological repeats of Tibetan sheep. H1, H2, and H3 represent three biological repeats of Hu sheep

### Differential expressed genes analysis

**Fig. 2:**
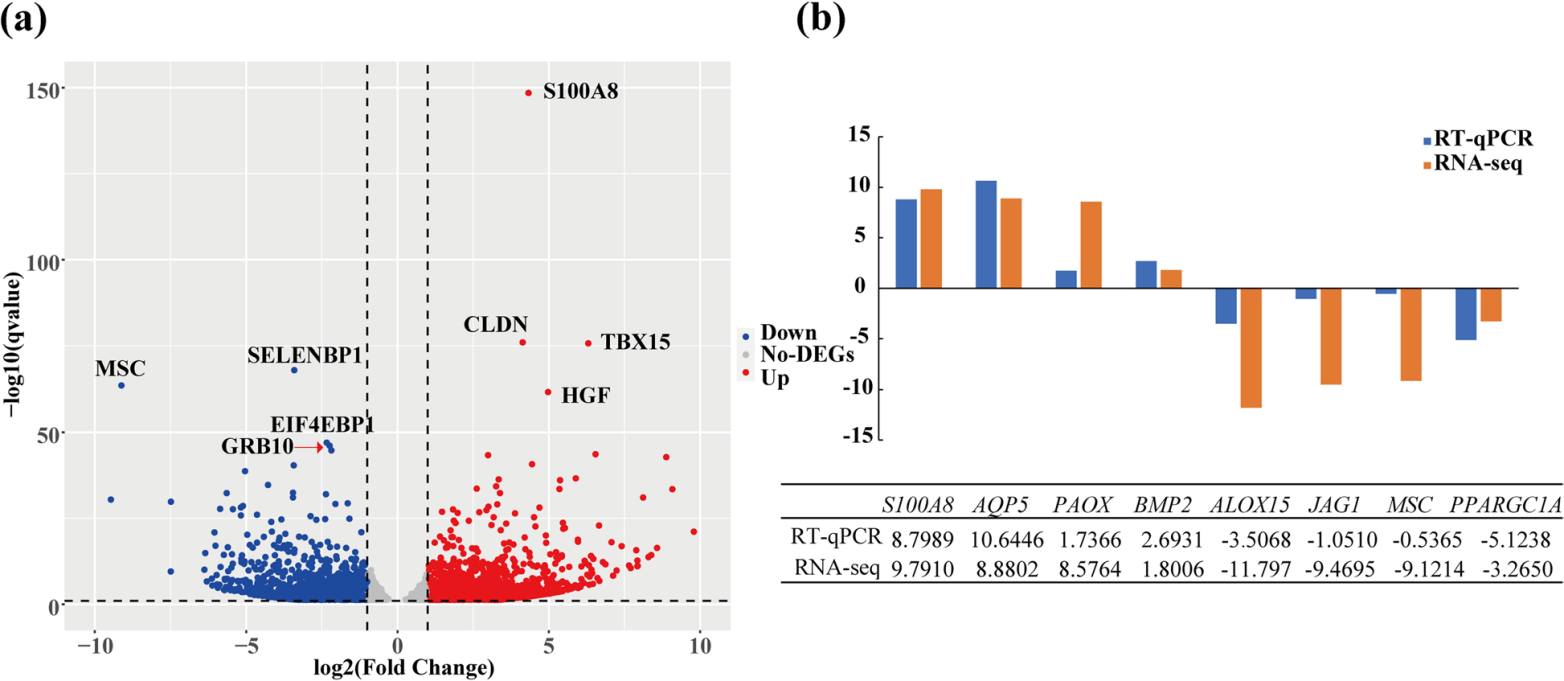
**a** The upregulated and downregulated genes in adipose tissue of Hu sheep tail. **b** RT–qPCR validation of 8 randomly selected genes identified by RNA-Seq analysis

RNA-Seq analysis of the two sheep breeds showed a total of 2108 genes detected using the reference genome. The genes with |FRKM|≥ 1.5 *and FDR* ≤ *0.01* were identified as DEGs, resulting in 1247 upregulated and 861 downregulated DEGs in the Hu sheep breeds. The top 8 differentially expressed genes involved in fat deposition were *S100A8*, *CLDN*, *TBX15* and *HGF*, which were upregulated in Hu sheep, while *SELENBP1*, *MSC*, *EIF4EBP4* and *GRB10* were downregulated in Hu sheep (Fig. 2, Table S1).

Eight DEGs were selected for RT–qPCR to validate our RNA-seq data. Among these DEGs, the trends of gene expression changes were consistent with the RNA-seq results (Fig. 2b).

### GO and KEGG enrichment analyses

**Fig. 3:**
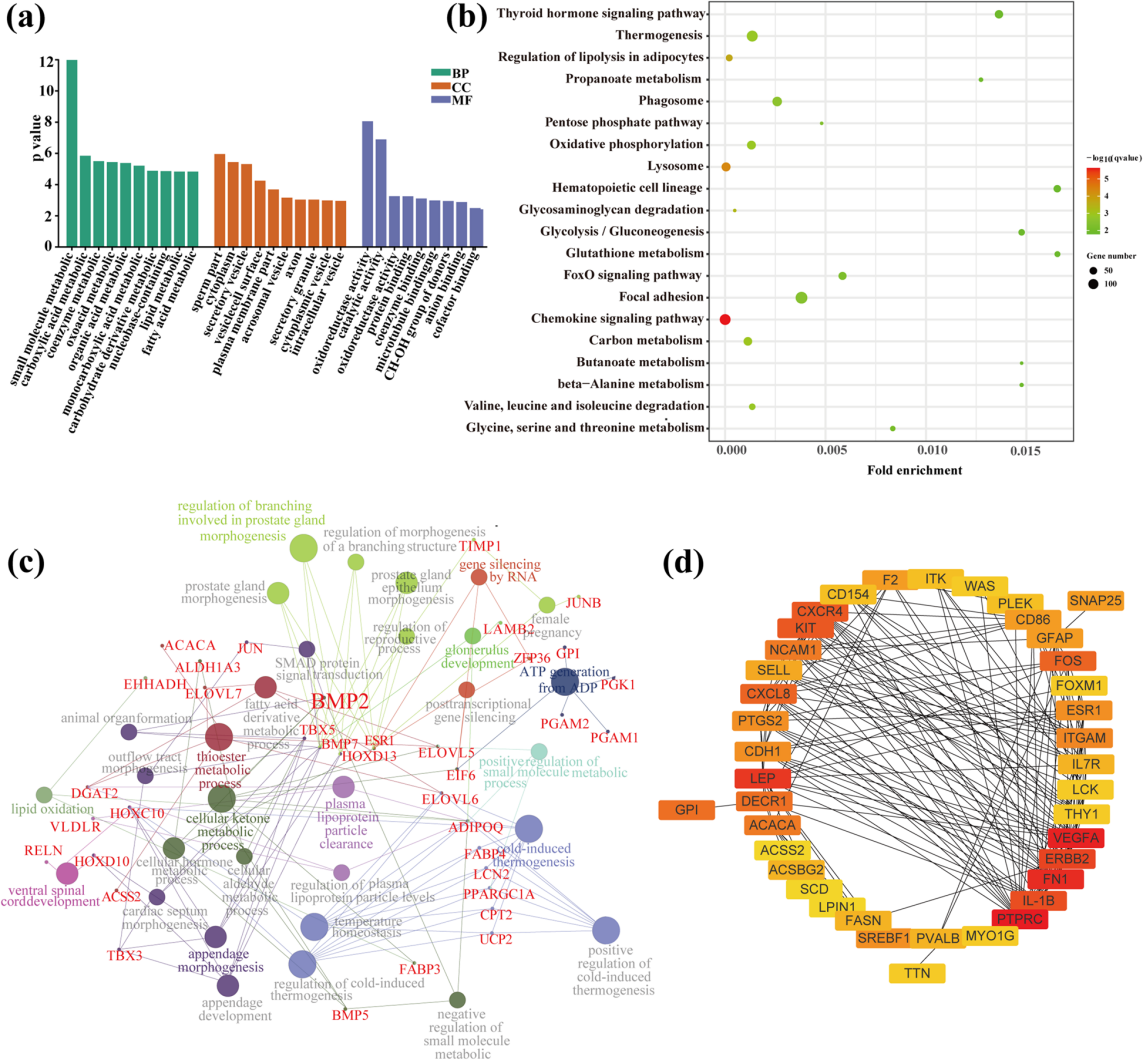
**a** GO enrichment for differentially expressed genes in adipose tissue of Hu sheep tail and Tibetan sheep; **b** KEGG enrichment for differentially expressed genes in adipose tissue of Hu sheep tail and Tibetan sheep; **c** Mechanism of fat deposition signalling pathway in differentially expressed genes; **d** The top 40 hub differentially expressed genes identified in PPI networks

GO and KEGG enrichment analyses were performed to obtain the biological relationships of the DEGs. The results of GO terms showed a nominal significance of *Q-value* < *0.01* in that 134 GO terms were enriched in the three groups (Fig. 3a). These included lipids metabolic process (GO:0,006,629), fatty acid metabolic process (GO:0,006,631) and cellular lipid metabolic process (GO:0,044,255). KEGG also identified 13 significant pathways (*Q-value* < *0.01*), including regulation of lipolysis in adipocytes (*Q-value* = 2.04E-04), carbon metabolism (*Q-value* = 1.13E-04), thermogenesis (*Q-value* = 1.35E-04) and glycine, serine, and threonine metabolism (*Q-value* = 8.35E-04) (Fig. 3b). These pathways were involved in fat metabolism or energy metabolism. ClueGO functional analysis of these potential DEGs also constructed a plausible pathway network for fat deposition in sheep [15]. A total of 219 terms were enriched. ClueGO analysis showed that most DEGs were regulated by fatty acid derivative metabolic processes (*P value*_Bonferroni_ = 0.0096), regulation of plasma lipoprotein particle levels (*P value*_Bonferroni_ = 0.0249) and lipid oxidation (*P value*_Bonferroni_ = 0.0294) (Fig. 3c). Furthermore, some pathways associated with cold stimulation were also enriched, such as regulation of cold-induced thermogenesis (*P value*_Bonferroni_ = 1.8618E-06), cold-induced thermogenesis (*P value*_Bonferroni_ = 1.8618E-06) and temperature homeostasis (*P value*_Bonferroni_ = 6.3048E-06).

To further determine the functional relationship to understand the DEGs, a PPI network formed interactions between upregulated and downregulated DEGs. According to node degree, we identified hub genes among these important DEGs. For better visualization, we reconstructed the interactors of the top 40 DEGs (Fig. 3d) using the cytohubba plug-in. *GPI*, *ACACA*, *ACSS2*, *TTN*, and *FASN* were upregulated in Hu sheep, and *WAS*, *LPIN1*, *GFAP*, and *FN1* were downregulated in Hu sheep.

### The effect of *BMP2* on adipogenesis induction

**Fig. 4:**
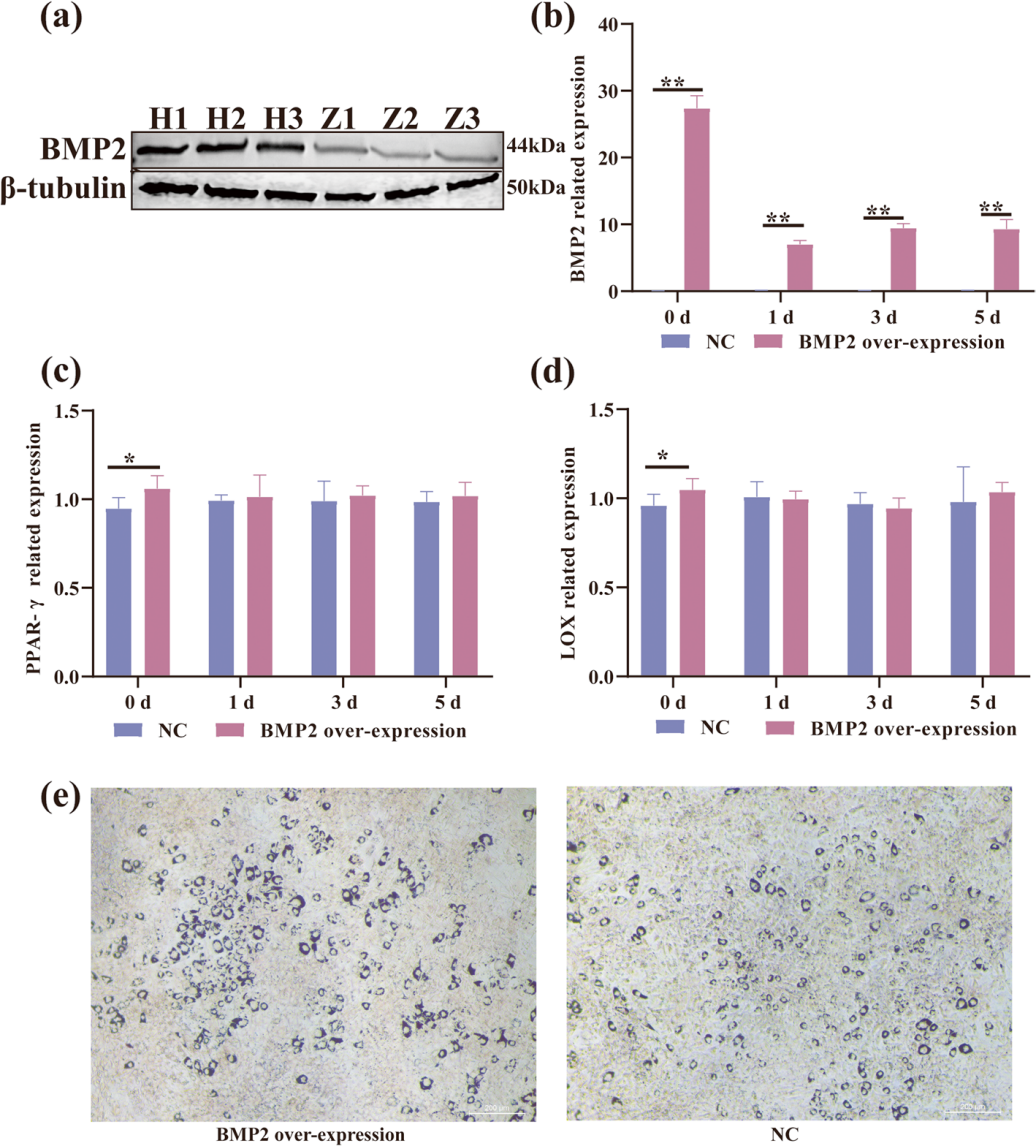
**a** The regulation of the protein level of BMP2 in adipose tissue of Hu sheep tail (H1, H2 and H3) and Tibetan sheep tail (Z1, Z2 and Z3). We cut 30-70 kDa of protein to transferred onto a PVDF membrane. **b** Relative expression of BMP2 in BMP2 over-expression and NC. **c** Relative expression of *PPAR-γ* in BMP2 over-expression and NC. **d** Relative expression of *LOX* in BMP2 over-expression and NC. **e** Oil red O staining when BMP2 was overexpressed in sheep preadipocytes

Based on our previous studies, we applied selection signal analysis to identify selection signals in sheep with different tail types [12]. These two methods found 43 candidate genes that may potentially be related to fat tail development, including *BMP2*, *HOXA11* and *PPP1CC,* which may play important roles in fat tail formation. Among these genes, *BMP2* is also strongly selected in the largest region by hapFLK [12]. Fat-tailed fixation is caused by a selective sweep near the retrotransposition hotspot on chromosome 13, and this diversity affects *BMP2* expression [14, 16]. We performed western blotting of *BMP2* in the tail fat of Hu sheep and Tibetan sheep, and the results showed that *BMP2* was highly expressed in Hu sheep (Fig. 4a). This was therefore selected for *BMP2* overexpression in preadipocytes. *BMP2* overexpression lentivirus transfected into preadipocytes (Fig. 4b), and after induced differentiation, *BMP2* overexpression also increased the mRNA levels of *PPAR-γ* and *LOX* when compared with NC at 0 d (*P value* < *0.05*) (Fig. 4c, d). There was no difference at 1, 3 and 5 days. Sheep preadipocytes on day 5 were stained with oil red O. Many small lipid droplets were stained red, and lipid rings were visible. The number of lipid drops in the *BMP2* overexpression group was greater than that in the NC group (Fig. 4e), which showed that fat deposition in the sheep tail was increased by *BMP2* overexpression.

## Discussion

Sheep tail fat deposition has a complex genetic regulation mechanism, which is determined by the environment and genes. Fat-tails are used to save energy in food plentiful seasons. During cold winters and in harsh environments, it provides the necessary energy to help sheep subsist [3]. Tail adipose tissue is an important tissue site for fat deposition in fat tail sheep. Research on the tail cutting of Mongolian sheep and Lanzhou large-tailed sheep found that the fat originally deposited in the tail was blocked by the tail fat metabolic pathway, and part of the fat was transferred and deposited in the rest of the body, mainly subcutaneous fat and intra-abdominal fat [17]. Therefore, the study of the fat tail is also an important material in the study of fat metabolism. Hu sheep are a short fat-tailed sheep breed and are also a good model to study fat deposition in the tail. Previous studies have mainly focused on the differential gene expression patterns or molecular genetic mechanisms among different sheep [1, 2, 8, 9, 18]. The mechanism by which mRNA regulates fat deposition is largely unknown.

In this study, a total of 2108 DEGs were identified as differentially expressed in Hu sheep and Tibetan sheep using RNA-seq. The purpose of this study was to assess the expression differences between fat-tailed and thin-tailed transcripts in sheep, which may contribute to a better understanding of the regulation of fat deposition metabolism in sheep. Through GO and KEGG enrichment analyses, we identified 134 and 13 pathways, respectively. DEGs were mostly directly or indirectly related to metabolic activity pathways, such as fatty acid metabolic processes, regulation of lipolysis in adipocytes and lipid metabolic processes [19]. This result implies that the molecular mechanism of fat deposition in the sheep tail is controlled by interactions that occur in a complex network of genes. Fat deposition in sheep tails may be regulated by multiple genes [20]. In this study, some upregulated DEGs were related to lipid metabolism, including *S100A8*, *BMP2*, *ADIPOQ*, *DGAT2*, *VLDLR*, *ELOVL6*, *GPI* and *ACACA*. *S100A8* plays an important role in the inflammatory responses of obese adipose tissue and contributes to the pathogenesis of obesity [21]. *ADIPOQ is* mainly secreted by adipose tissue and is a kind of adipocytokine [22]. *ADIPOQ* is also a major component in lipid metabolism by inhibiting lipid synthesis and promoting fatty acid oxidation [22, 23]. This gene was highlighted in transcriptome analysis of sheep fat deposition [1, 8, 24]. In addition, genetic variation in *ADIPOQ* is associated with fat deposition in pigs [25] and meat marbling in cattle [26]. *DGAT2* is crucial in catalysing the production of triacylglycerol [27]. *DGAT2* was also identified to affect fat deposition in sheep [8] and pigs [28]. *VLDLR* is involved in triglyceride metabolism [29] and is associated with fat deposition in pigs [9]. *ELOVL6* plays an important role in controlling the overall balance of fatty acid components [30] and is also found in pigs [30] and sheep [2]. *ACACA* is a key regulator of lipogenesis in the adipose tissue of animals [31, 32]. *GPI* acts in the glucose pathway into pentose phosphate and produces NADPH, which is necessary for lipid metabolism [33, 34]. In our study, *ACACA* and *GPI* were selected in the PPI network, which may reveal that upregulation of these DEGs may induce fat deposition in the Hu sheep breed. In a previous study, we also identified *BMP2* as playing an important role in sheep tail formation [12, 13]. A previous study also showed that *BMP2* promotes the expression of *PPAR-γ* in preadipocytes and promotes the differentiation of preadipocytes from sheep [35]. In our study, the result of oil red O at 5 days showed that fat deposition in the sheep tail was increased by *BMP2* overexpression. This finding confirms that *BMP2* promoted differentiation of preadipocytes. The results are consistent with previous results [35]. Yuan et al. also showed that *BMP2* was associated with fat deposition [12]. Moreover, *LOX* is a bona fide downstream target gene of the BMP signalling pathway [36]. During adipocyte lineage commitment, *LOX* is induced by *BMP2/4* [37]. These results were similar to our studies. *BMP2* overexpression in preadipocytes promoted *PPAR-γ* and *LOX* expression at 0 days. *BMP2* potentially induces adipogenesis through *LOX* in preadipocytes. The present results failed to demonstrate the exact mechanism of *LOX* expression promoted by *BMP2*. Future studies are needed to determine the mechanism by which *LOX* is promoted by *BMP2* in preadipocytes to pinpoint the true induction of adipogenesis.

Furthermore, GO terms revealed DEGs enriched in cold-induced thermogenesis. The cold-induced thermogenesis pathway is important in the regulation of adipogenesis and lipid metabolism [38, 39]. Cold exposure activates brown fat to stimulate lipolysis but also leads to an increase in fatty acid synthesis within tissues [40]. Interestingly, *CPT2*, *LCN2*, *PPARGC1A*, *UCP2* and *HOXC10* were enriched in GO terms related to thermogenesis. Among these genes, *CPT2* is important for fatty acid oxidation [41] and is widely expressed as an energy-producing tissue [42]. *CPT2* was also identified with RNA-seq in Lori-Bakhtiari and Zel sheep [2]. *LCN2* may act as an anti-obesity agent by upregulating thermogenic markers, leading to browning in white adipose tissue [43]. *PPARGC1A* plays a role in stimulating mitochondrial biogenesis and regulating glucose and fatty acid metabolism [44, 45]. Moreover, *UCP2* is well known to be involved in resistance to diet-induced obesity [46]. Previous studies have proven that HOX genes are associated with anterior to posterior vertebrate axial morphology [17, 18]. The caudal regions of short thin-tailed and fat-tailed sheep differ significantly, characterized by significant changes in fat mass, which may largely depend on the number of vertebrate caudal vertebrae and require further study [18]. In our study, *HOXC12*, *HOXA6*, *HOXA4*, *HOXD13*, *HOXB2* and *HOXD10* were identified as DEGs in Hu sheep and Tibetan sheep. In addition, *HOXA6*, *HOXB2*, *HOXD10*, *BMP7* and *TBX15* were enriched in embryonic skeletal system development terms. *BMP7* can induce brown adipogenesis, and overexpression in adipose tissue induces white adipogenesis [47]. *TBX15* is involved in encoding phylogenetically conserved transcription factors that regulate developmental processes and plays a vital role in regulating muscle metabolism and glycolytic fibre identity [48, 49].

## Conclusion

In this study, transcriptome analysis was first performed in Hu sheep and Tibetan sheep. Some GO terms and pathways associated with lipid deposition were identified. PPI network analysis showed that DEGs were significantly involved in lipid metabolism. These DEGs may be important in fat tail metabolism in sheep. Among these DEGs, *BMP2* potentially induces adipogenesis through *LOX* in preadipocytes. The exact mechanism also needs to be studied in further investigations. In short, these findings provide a new theoretical basis for the study of the mechanism of fat-tailed sheep.

## Materials and methods

### Animals and sample collection

Six male sheep (1.5 years old with approximately equivalent weights) from each purebred Hu sheep (H1, H2, H3, Yongdeng, Gansu) and Tibetan sheep (Z1, Z2, Z3, Yushu, Qinghai) were used in this study. Adipose tissue from tails of the sheep were collected and washed with 0.9% NaCl and then frozen in liquid nitrogen until RNA was extracted.

### RNA extraction and sequencing

Total RNA was isolated from fat tissue samples using the Standard Sensitivity RNA Analysis Kit (15 nt) according to the manufacturer’s instructions. RNA was quantified, and the purity of the samples was determined using the Fragment Analyser to evaluate the 28S/18S ratio. All samples with an OD_260/280_ ratio greater than 1.9 and 28S/18S ratio > 1.2 were sequenced by BGI (Beijing, China). RON and RNA integrity number (RIN) were measured, and only RIN/RQN ratios greater than 7.5 were used for cDNA library construction. Then, cDNA libraries were sequenced on the BGISEQ-500 platform by a paired-end strategy. The raw data have been deposited in the Sequence Read Archive with the indicated accession codes (BioProject ID: PRJNA792697).

### Quality control and mapping genome to identified DEGs

We carried out quality control on raw data to detect common issues using the SOAPnuke v1.4.0 program [50]. Then, raw reads were trimmed with Trimmomatic to remove adapter sequences and poly-N and low-quality sequence reads to obtain clean reads [2, 51]. Clean reads were saved in fastq format. Clean reads were processed using HISAT v2.1.0 [52], and the clean reads were aligned to *Ovis_aries* (Oar_v3.1). Moreover, using Bowtie v2.2.5, we built an index of the reference genome [53], and the level of expression was calculated using RSEM [54]. In addition, principal component analysis (PCA) was performed using SARTools software [55]. According to the gene expression patterns, the samples were clustered, and the degree of similarity/difference between the gene expression profiles was detected [2].

### Functional analysis and PPI network construction

Phyper function in R performed gene set enrichment analysis in up- or downregulated genes. The significant enrichment of Gene Ontology (GO) functional terms with DEGs that showed differential expression was analysed (*P value* ≤ *0.01*) [56]. Furthermore, the significantly DEGs were also enriched in the Kyoto Encyclopedia of Genes and Genomes (KEGG) database (*P value* ≤ *0.05*) [57]. Among these DEGs, protein–protein interactions (PPIs) were considered in the STRING database (https://cn.string-db.org/) [58].

### Isolation of preadipocytes from adipose tissue of Hu sheep and transfection of BMP2 overexpression

The *BMP2* overexpression lentivirus was constructed as described by Lu [14]. An empty lentivirus vector was used as the negative control (NC) [14]. Preadipocytes were isolated from Hu sheep tail fat (70-day-old foetus). Primary preadipocytes were cultured in vitro by collagenase digestion. The cells were incubated in complete medium at 37 ℃ for two days, and the cells were almost all adherent to the wall. Cells were cultured in a plate with 1000 µl medium (5% FBS, 1% PS) in 6-well plates, and the titration of lentiviruses was MOI = 100 of the final construct together with BMP2 overexpression in triplicate for 24 h. Then, the cells were transferred to new complete medium (10% FBS, 1% PS). When the cells showed contact inhibition, the induction differentiation medium (complete medium + 0.5 mM isobutylmethylxanthine + 10 mg/mL insulin + 1 µM dexamethasone) was changed for 2 days. The final cells were cultured in maintenance differentiation medium (complete medium + 10 mg/mL insulin) for 2 days [59]. Cells were set as the first day when cultured with differentiation medium. RNA was extracted from cells with BMP2 overexpression and NC at various times (0, 1, 3 and 5 days).

### Oil red O staining

Oil red O dye (Solarbio, China) and distilled water at a ratio of 3:2 was used to filter the mixture. Cells differentiated for 5 days were washed twice with PBS and fixed with 4% paraformaldehyde for 20 min. Then, oil red O dye was added, and the cells were incubated for 15 min, washed with distilled water 2–3 times and observed and photographed under a microscope.

### RT–qPCR and western blot

Reverse transcription was performed according to the instructions of the Primer Script II 1st strand kit (Takara, China). The primers were designed by Primer 5.0 (Table S2). β-actin was used as the reference gene. Three biological replicates and triplicate technical replicates were obtained. The reaction and calculation were described by Jin [60].

Fat tissues were extracted from tails of Hu sheep (H1, H2 and H3) and Tibetan (Z1, Z2 and Z3) and 1 ml RIPA lysis buffer and 1 mM PMSF (Beyotime, Shanghai, China) were added to obtain the total proteins; thus, the protein concentrations were measured with the BCA method (Beyotime, China). Proteins were separated on 10% SDS–PAGE and cut 30–70 kDa of protein to transferred onto a PVDF membrane (Millipore, USA). The membrane was sealed with quick sealing fluid (Lablead, China) and washed with TBST (Solarbio, China) three times. Proteins were detected with rabbit monoclonal anti-β-tubulin (50 kDa, Proteintech, USA) and rabbit monoclonal BMP2 (44 kDa, Proteintech, USA). The reaction band was developed by using enhanced chemiluminescence (Epizyme, China) and images of the PVDF membrane were recorded with a JP-K600 imaging system (JiaPeng, China).

## Supplementary Information

**Additional file 1. **

**Additional file 2: Table S1.** The top 8 most significantly affected differentially expressed genes in Hu sheep and Tibetan sheep. **Table S2.** Primers used in this study for RT-qPCR.

## Data Availability

All the RNA-seq reads have been deposited in the Sequence Read Archive (https://www.ncbi.nlm.nih.gov/sra) with the accession codes (BioProject ID: PRJNA792697).

## Abbreviations

DEGs: Differentially expressed genes
GO: Gene Ontology
KEGG: Kyoto Encyclopedia of Genes and Genomes
FPKM: Fragments Per Kilobase per Million
*BMP2*: Bone Morphogenetic Protein 2
*PPAR-γ*: Peroxisome Proliferator Activated Receptor γ
*LOX*: Lysyl Oxidase
