# Supplementary figures and images for: Transcriptome study digs out BMP2 involved in adipogenesis in sheep tails
Source: BMC Genomics. 2022 Jun 21;23:457. doi: 10.1186/s12864-022-08657-8 (PMC9210821; doi:10.1186/s12864-022-08657-8)

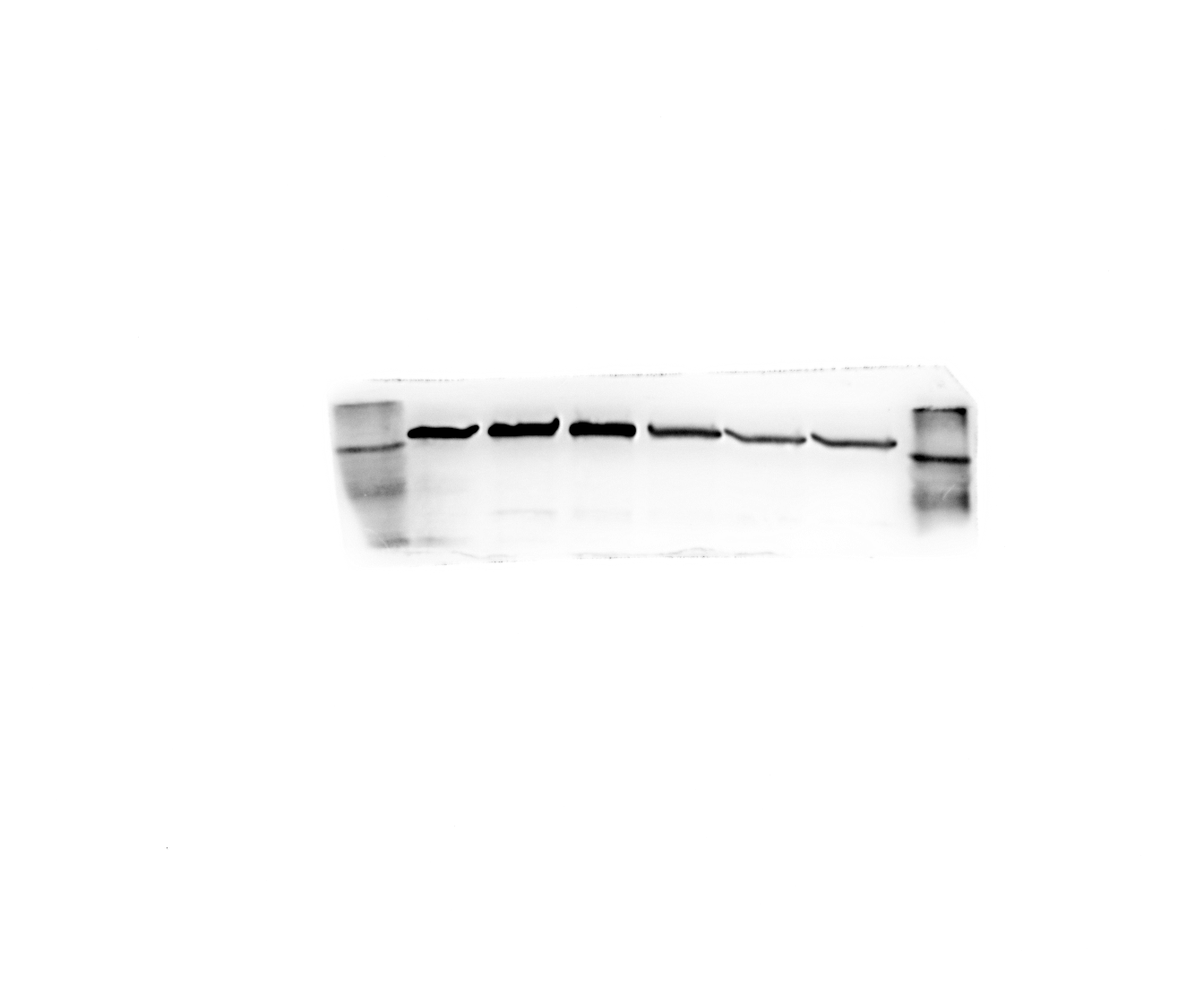

Supplement: Supplementary file 1 — Additional file 1. [file 12864_2022_8657_MOESM1_ESM.zip › BMP2-images_20000ms.tif]

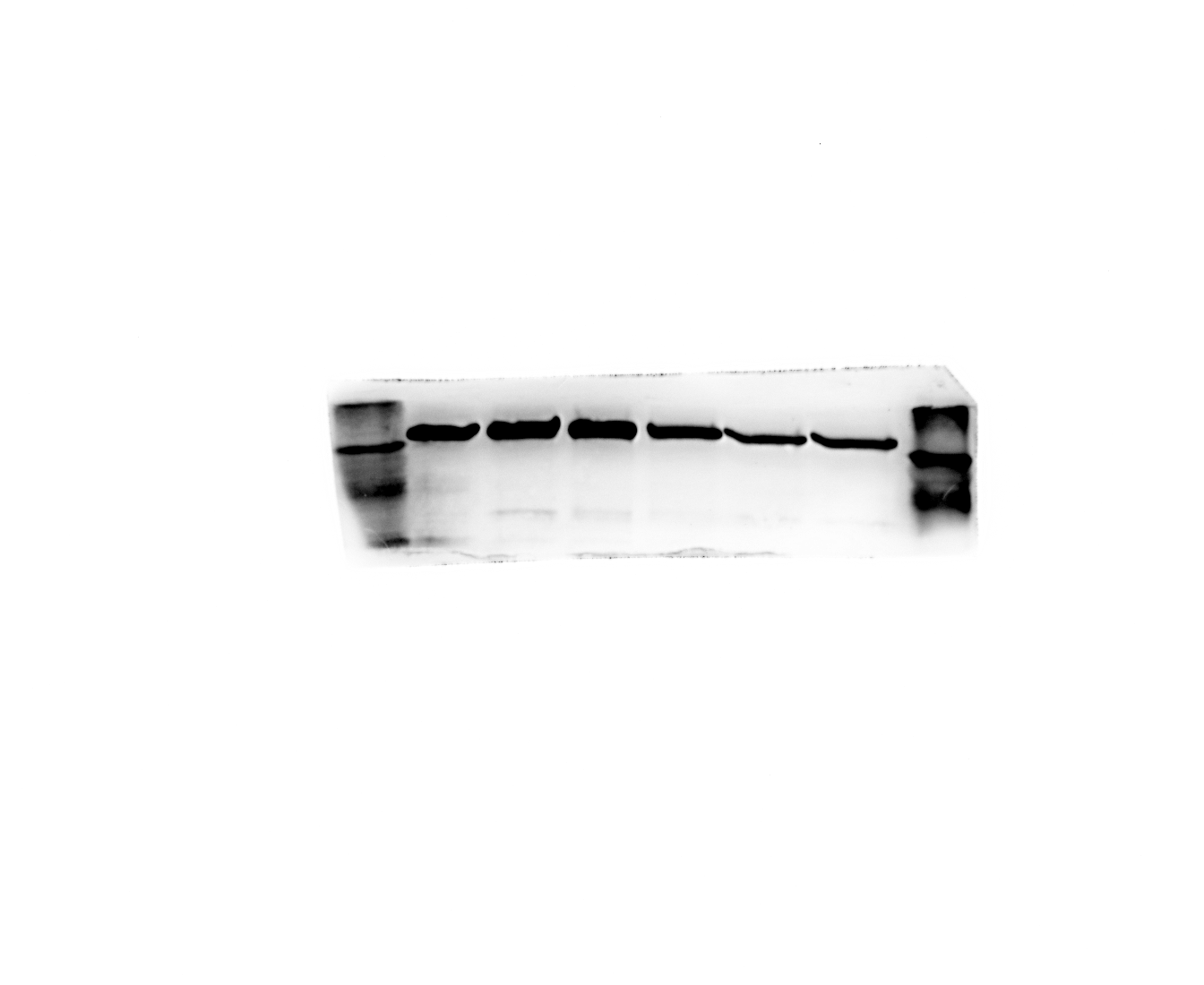

Supplement: Supplementary file 1 — Additional file 1. [file 12864_2022_8657_MOESM1_ESM.zip › BMP2-images_40000ms.tif]

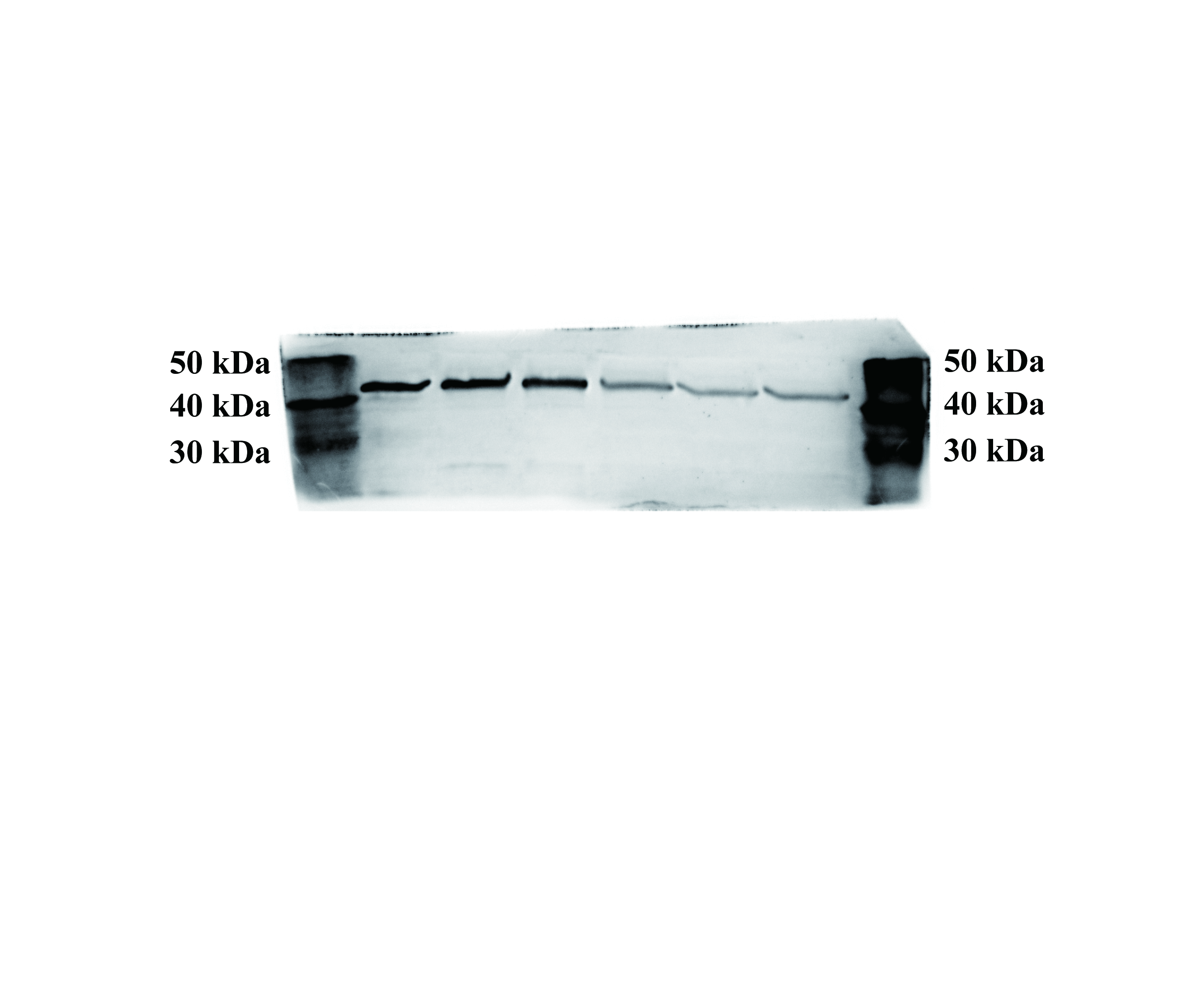

Supplement: Supplementary file 1 — Additional file 1. [file 12864_2022_8657_MOESM1_ESM.zip › BMP2-western blot.tif]

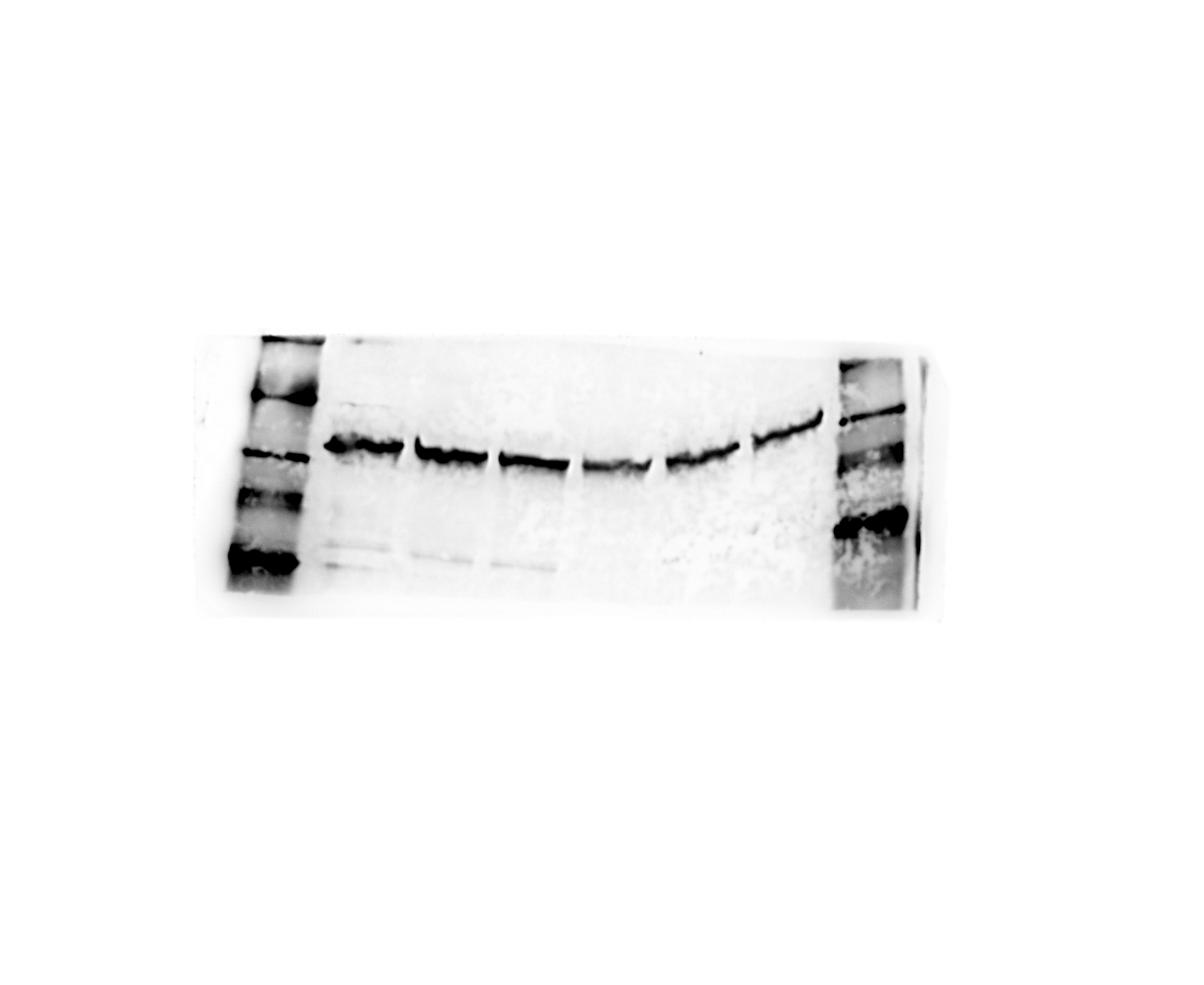

Supplement: Supplementary file 1 — Additional file 1. [file 12864_2022_8657_MOESM1_ESM.zip › ß-tubulin-images_20000ms.tif]

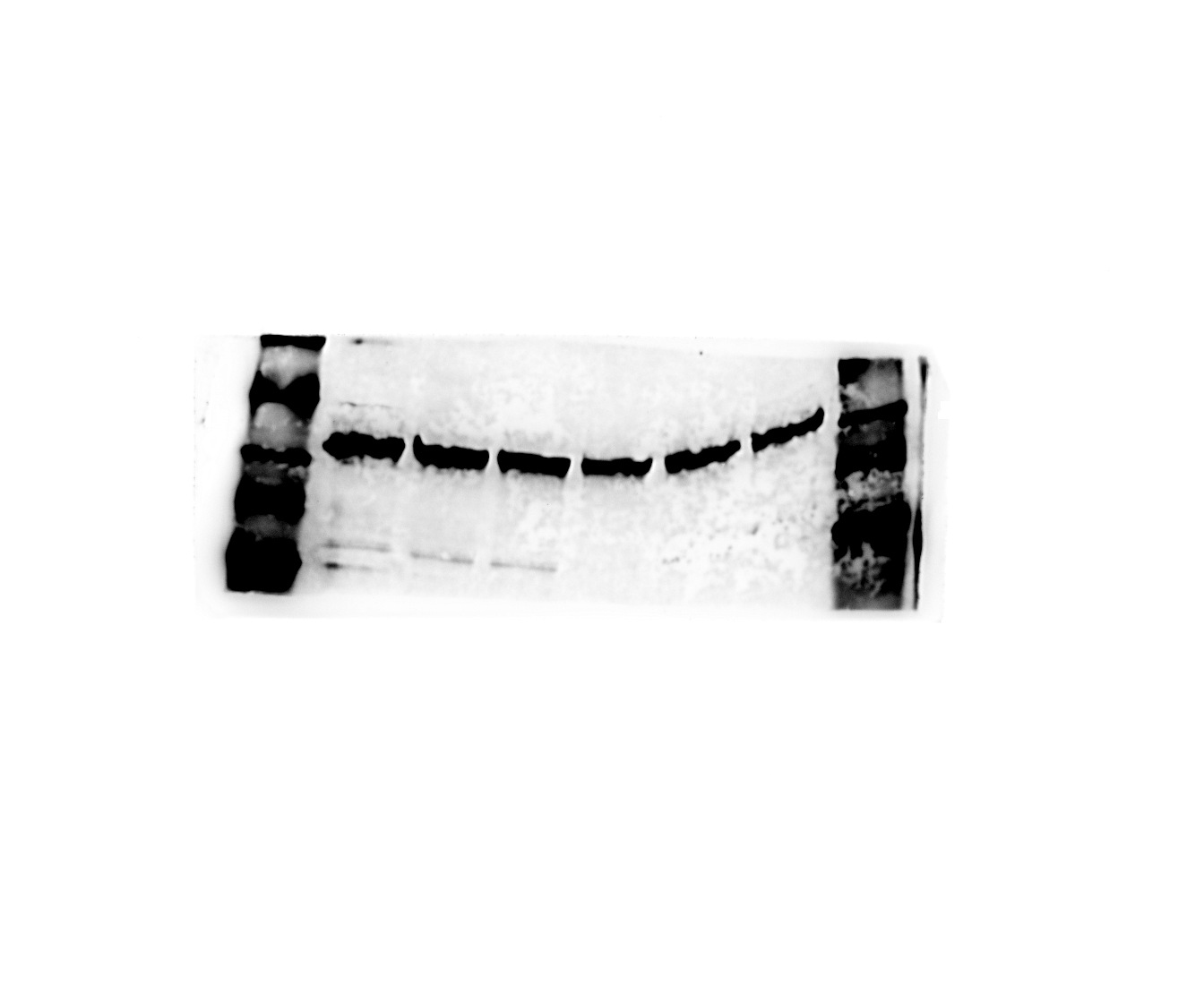

Supplement: Supplementary file 1 — Additional file 1. [file 12864_2022_8657_MOESM1_ESM.zip › ß-tubulin-images_40000ms.tif]

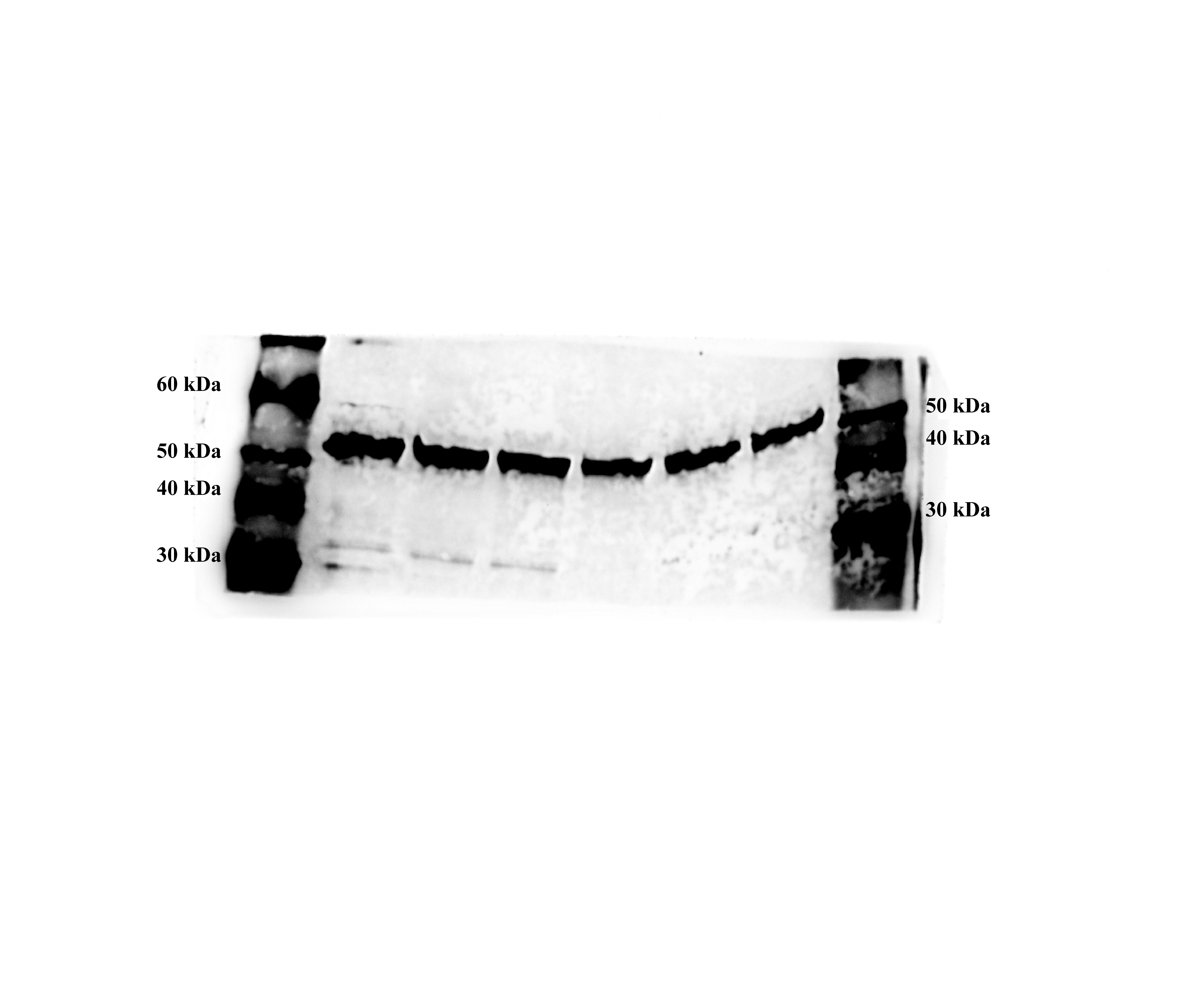

Supplement: Supplementary file 1 — Additional file 1. [file 12864_2022_8657_MOESM1_ESM.zip › ß-tubulin-western blot.tif]
